# Data hazards in synthetic biology

**DOI:** 10.1093/synbio/ysae010

**Published:** 2024-06-21

**Authors:** Natalie R Zelenka, Nina Di Cara, Kieren Sharma, Seeralan Sarvaharman, Jasdeep S Ghataora, Fabio Parmeggiani, Jeff Nivala, Zahraa S Abdallah, Lucia Marucci, Thomas E Gorochowski

**Affiliations:** Jean Golding Institute, University of Bristol, Bristol, UK; BrisEngBio, University of Bristol, Bristol, UK; School of Psychological Science, University of Bristol, Bristol, UK; School of Engineering Mathematics and Technology, University of Bristol, Bristol, UK; School of Biological Sciences, University of Bristol, Bristol, UK; BrisEngBio, University of Bristol, Bristol, UK; School of Biological Sciences, University of Bristol, Bristol, UK; BrisEngBio, University of Bristol, Bristol, UK; School of Biochemistry, University of Bristol, Bristol, UK; School of Pharmacy and Pharmaceutical Sciences, Cardiff University, Cardiff, UK; Paul G. Allen School of Computer Science and Engineering, University of Washington, Seattle, WA, USA; School of Engineering Mathematics and Technology, University of Bristol, Bristol, UK; BrisEngBio, University of Bristol, Bristol, UK; School of Engineering Mathematics and Technology, University of Bristol, Bristol, UK; BrisEngBio, University of Bristol, Bristol, UK; School of Biological Sciences, University of Bristol, Bristol, UK

**Keywords:** data hazards, data science, AI, synthetic biology, ethics

## Abstract

Data science is playing an increasingly important role in the design and analysis of engineered biology. This has been fueled by the development of high-throughput methods like massively parallel reporter assays, data-rich microscopy techniques, computational protein structure prediction and design, and the development of whole-cell models able to generate huge volumes of data. Although the ability to apply data-centric analyses in these contexts is appealing and increasingly simple to do, it comes with potential risks. For example, how might biases in the underlying data affect the validity of a result and what might the environmental impact of large-scale data analyses be? Here, we present a community-developed framework for assessing data hazards to help address these concerns and demonstrate its application to two synthetic biology case studies. We show the diversity of considerations that arise in common types of bioengineering projects and provide some guidelines and mitigating steps. Understanding potential issues and dangers when working with data and proactively addressing them will be essential for ensuring the appropriate use of emerging data-intensive AI methods and help increase the trustworthiness of their applications in synthetic biology.

## 1. Introduction

Synthetic biology has seen a rapid expansion in the use of data-centric approaches for biological design over the past decade (1–3). By employing methods like deep learning trained on the vast biological datasets that are now becoming available (4–8), researchers can predict the behavior of complex biological systems and design new biological parts and circuits with unprecedented precision and control (9–12). While these advances have the potential to revolutionize various aspects of biotechnology, they also present a number of challenges and potential risks that require careful consideration.

One of the primary challenges in this area is the quality and reliability of the data used to build and validate the models (13). The accuracy and utility of data-centric models depend heavily on the underlying data that are used to build them. Data can be prone to errors, biases, and inconsistencies (14–16). As a result, models based on flawed or incomplete data can lead to unexpected results, such as the creation of a genetic circuit or synthetic organism with unpredictable behavior, or the inference of erroneous biological insights that hamper progress in fundamental and applied research.

The increasing complexity of data-centric approaches in synthetic biology also raises concerns about their interpretability and transparency (17). As models become more intricate and incorporate larger datasets [e.g. large neural networks (4, 9, 12) or whole cell models (18)], it becomes increasingly difficult for researchers to understand the underlying mechanisms driving their predictions. This lack of transparency hinders efforts to validate and improve these models, which is essential for ensuring their safe and responsible application.

The potential misuse of data-centric approaches in synthetic biology poses a further significant risk. The ease of access to data science tools may enable nefarious actors to develop harmful biological agents for purposes such as bioterrorism or to disrupt ecological systems intentionally. In addition, the rapid dissemination of synthetic biology techniques and knowledge, combined with a culture that fosters collaboration and innovation, could also increase the risk of an accidental (or willing) release of biological agents with unforeseen (or underestimated) consequences. Many of the models themselves also pose a significant environmental impact that is often unseen, with vast amounts of computing resources and electricity required to generate predictions or train models (19).

More broadly, the increased use of data-centric approaches across all science and technology has also led to many ethical oversights and mistakes (20–22). These have often appeared avoidable retrospectively, with the general public and researchers from other disciplines raising alarms before the tools in question were deployed (23). However, data science and AI practitioners, who have the power to make decisions to improve the positive impact of their research, continue to find it difficult to engage with ethics work (24–26). In many cases they are disincentivized to do so, they often are not supported or trained appropriately, and many feel that ethics frameworks are either vague, unstructured, and difficult to apply, or worse still, just box ticking exercises that outsource the ethical judgment to committees who don’t always understand what their research can do. Some initiatives attempt to overcome these issues. For example, the “AI Blindspot” project (https://aiblindspot.media.mit.edu) aims to proactively uncover potential oversights as an AI project is developed, highlighting potentially harmful unintended consequences. While hugely valuable for improving the safety of AI research, existing frameworks like this are typically focused purely on impacts that would directly affect humans. The potential of AI systems to harm the environment and wider ecosystems is often neglected, but of paramount concern when dealing with AI applied to engineered biology.

The use of data-centric approaches in synthetic biology offers exciting prospects for advancing our ability to engineer biological systems. However, it is crucial to proactively acknowledge and address the challenges, risks, and ethical considerations associated with these new methods. In this work, we present a community developed assessment framework called “Data Hazards” that aims to address some of these difficulties by supporting the more thorough consideration of potential data-related hazards that might exist as a project develops. While the framework is field agnostic, here we develop several extensions specific for synthetic biology applications, present two case studies to illustrate how the framework might be applied to protein design and whole-cell modeling tasks, and end by discussing potential mitigation strategies for issues that could arise. This work contributes to the ongoing conversations about responsible innovation in synthetic biology (27, 28) and the challenges that applications of data science bring to the field.

## 2. Materials and methods

### 2.1 Data hazards resources

**Figure 1.**
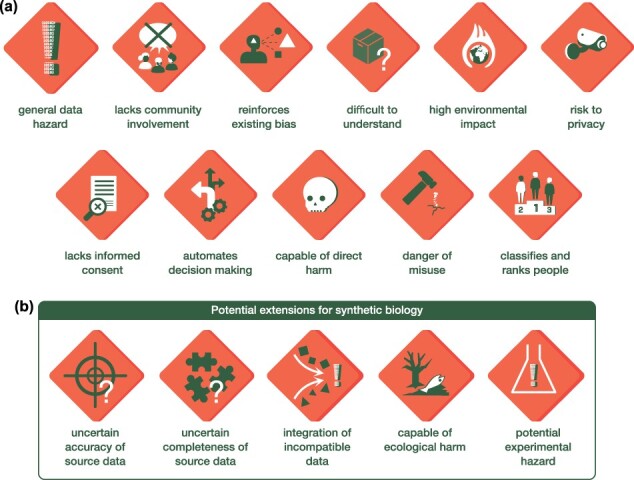
Overview of the data hazard labels. (a) Standard set of data hazard labels. Each label has been designed to clearly capture a core area of potential concern. One or many labels may apply to a piece of data science research. (b) Potential extensions to data hazard labels that address challenges common in synthetic biology research.

The Data Hazard labels (Figure 1a) are generally applied to projects through workshops or self-assessment and, following this, the label-specific safety precautions and cross-label hazard mitigation resources are used to identify potential interventions to mitigate risks. The Data Hazards website provides a user-guide for these common uses of the labels. This includes teaching materials (e.g. lesson plans and printable Hazard labels), workshops (e.g. checklists, timings, e-mail and feedback templates, slides, facilitating tips), a self-assessment guide, hazard mitigation resources, guides to displaying the labels, and finally case studies from users. All data hazard labels are available as Supplementary Information.

## 3. Results

### 3.1 A community framework for assessing data hazards

Data Hazards is an open-source, Creative Commons Attribution (CC-BY) licensed resource that aims to support data science and AI practitioners in identifying the broad risks associated with their work such as environmental concerns, misuse and algorithmic bias, and allows the to consider practical processes and activities to mitigate these (29). The resource is centered around a community-generated and evolving vocabulary of ethical risks, presented as “hazard labels” that are inspired by chemical warning signs (Figure 1a). Each label consists of an image, name, description, examples of where it applies, and safety precautions (Table 1). These act to facilitate interdisciplinary conversations and individual reflection and it is expected that they will lead to mitigating actions to address any issues raised. Without such safeguards, these labels could cause more harm than good, acting as “attention hazards” in their own right. Hazard labels associated with a project and mitigating actions can be displayed in posters, theses, ethical considerations sections of conference presentations and papers, or simply used as part of an internal process to identify necessary safeguards to improve the quality of research outputs. The hazard labels are relevant to any project that uses data, statistics, algorithms, machine learning, or AI, and have been applied to diverse projects spanning natural language processing in social media (30, 31), molecular modeling of molecules in neurons (32), and the integration of medical data sets (29, 33). Furthermore, by extending an existing safety framework that experimental scientists are already familiar with, and which covers both human and environmental impacts, we believe the barrier to adoption is lowered. When designing experiments in a laboratory, chemical hazards are assessed, and safeguards put in place; something that we feel should extend to the AI tools developed to support such research.

**Table 1.** Descriptions of data hazards with synthetic biology examples

Data hazard	Description	Synthetic biology examples	Potential safeguards
General data hazard	Data science is being used and leading to negative outcomes. This hazard applies to all data science research outputs.	All areas that make use of data science approaches.	Proactively explore potentially negative applications and implement mitigating actions.
Lacks community involvement	Technology is being produced without sufficient input from the community it is designed to serve.	Proprietary ML-based algorithms developed to support a synthetic biology based therapeutic with no Patient and Public Involvement and Engagement (PPIE).	Engage with community stakeholders through consultations and participatory design processes.
Reinforces existing bias	Reinforces unfair treatment of individuals and groups. This may be due to input data, algorithm or software design choices, or society at large.	Focus on data collection for a limited set of model organisms. May mean our understanding and models do not translate to biology at large and lead to poor decisions when engineering non-model species.	Apply algorithms to detect bias in datasets and model outputs, helping guide new data collection/generation to alleviate found biases.
Difficult to understand	Danger that the technology is difficult to understand. This could arise due to a lack of interpretability (e.g. neural nets), lack of documentation, or problems with implementation details that are difficult to spot.	Deep learning models of gene regulatory sequence and proteins. Large-scale models of cellular processes (e.g. whole-cell models, metabolic models, regulatory models)	Use standardized data formats (e.g. SBOL) and seek domain expertise to apply explainable AI approaches.
High environmental impact	Methodologies are energy-hungry, data-hungry (requiring increasing amounts of computation), or require special hardware that require rare materials and resources that are non-sustainable.	Large deep-learning-based models require huge amounts of compute for training and often significant compute for prediction, which typically has a hidden environmental impact. Similarly whole-cell models can take days to run and generate huge data sets that require significant storage.	Explore the use of surrogate modeling to reduce computational resources required, optimize code and hardware used.
Risk to privacy	Possible risk to the privacy of individuals whose data is processed.	Engineering of personalized medicine applications (e.g. CAR T cell engineering).	Anonymize data where possible.
Lacks informed consent	Datasets or algorithms use data which have not been provided with the explicit consent of the data owner/creator. These type of data often lack other contextual information, which can also make it difficult to understand potential biases.	Bioprospecting studies of large genomic data bases often make use of sequenced samples where consent of local people may not have been given.	Develop clear guidelines for obtaining informed consent and ensure transparency in data usage.
Automates decision-making	Automated decision-making can be hazardous in many different ways. Important to ask: whose decisions are being automated, what automation can bring to the process, and who benefits or is harmed by this automation?	Increasing use of automation and design of experiment approaches when screening libraries and performing complex laboratory tasks. Errors in data could result in poor decisions being automatically made.	Identify areas where decisions are being automated and adapt existing safety frameworks to increase testing/validation of design choices, prior to deployment.
Capable of direct harm	The application area of this technology means that it is capable of causing direct physical or psychological harm to someone even if used correctly.	Many areas of synthetic biology have dual-use (e.g. toxin production, synthetic viruses, etc.)	Assess level of harm and ensure sufficient containment is in place to avoid harm.
Danger of misuse	There is a danger of misusing the algorithm, technology, or data collected.	Synthetic biology often has dual-use and considering new-to-nature biological parts and systems can have difficult to predict unintended consequences (e.g. gene drives, toxin production, engineering of viruses).	Ensure thorough testing of models prior to release including the identification of potential “emergent abilities” in neural network-based generative models.
Classifies and ranks people	Ranking and classifications of people should be handled with care. We should ask what happens when the ranking/classification is inaccurate, when people disagree with how they are ranked/classified, as well as who it serves and how it could be gamed.	Less common in synthetic biology, but may become an issue if personalized medicine becomes established.	Seek engagement with society about how classifications might cause negative outcomes and aim to build broader agreement on how issues are best handled.

The project is managed through a website (https://datahazards.com), which houses the most up-to-date information regarding the aims and origin of the project, how data hazards can be best used, hints and tips for running workshops or self-assessing your own projects, options for contributing to the project, upcoming events, and examples of data hazard label use. The entire website and the associated resources are all stored in a public GitHub repository to allow for versioning control of all elements as the project develops.

### 3.2 Data hazards specific to synthetic biology research

The ability for data science to be applied to biological design means that all existing data hazards could potentially be applicable to synthetic biology research. But does synthetic biology bring further hazards to the table? We assessed some of the core challenges faced when using data-centric approaches to engineer biology and found five new data hazards that, while being relevant to synthetic biology, also touched upon key aspects of biological data more broadly.

Two such hazards relate to the nature of data typically collected from biological systems. Firstly, available biological datasets often have high levels of uncertainty associated with their measurements and may also be incomplete, providing only a limited picture of the underlying system. Both of these difficulties stem from biological processes being challenging to measure due to their complexity and dynamic nature, as well as an inability to observe these processes directly, meaning that proxies are commonly used (e.g. fluorescent reporter proteins used to measure gene expression). These factors result in inaccurate or incomplete datasets, which may have significant consequences when applying data science methods without an understanding of these limitations.

The interdisciplinary nature of synthetic biology can also lead to risks, as data of different types and from different sources may need to be integrated as part of data science pipelines. Furthermore, reproducibility of results across the life sciences remains a major challenge, and while there are efforts to improve the situation through the use of calibrants (34, 35) and minimal information standards (36, 37), large variations in measurements of even identical biological processes between different labs means that data scientists need to be keenly aware of possible incompatibilities in the data they use (e.g. measurements in different units). Perhaps even more difficult to catch are genetic differences in supposedly identical cell lines (38, 39), batch-to-batch variation in reagents (e.g. chemicals and media) (40), or the unintended variation in environmental factors when repeating experiments performed by other labs. Such information is often not captured during experiments and places questions over the quality and validity of the data produced and can potenitally impact downstream uses (e.g. for parameter fitting during modeling).

Finally, while the existing data hazard “capable of direct harm” (Figure 1a) captures impacts on other human-beings, synthetic biology opens up the potential for harm to be caused to other organisms and ecosystems more broadly [e.g. gene drives (41)], as well as the opportunity for experimental hazards that arise in the laboratory, but stem from data informed decisions with unexpected consequences (e.g. the accidental design of a new-to-nature enzyme that catalyzes an unknown ecologically harmful reaction).

For each of these cases, we developed new hazard labels that aim to capture their core features and act as an extension to the current library and recommendations (Figure 1b; Table 2; Supplementary Material).

**Table 2.** Descriptions of additional data hazards relevant for synthetic biology

Data hazard	Description	Synthetic biology examples	Potential safeguards
Uncertain accuracy of source data	The accuracy of the underlying data is not known and so its use may lead to erroneous results or introduce bias.	Metabolic modeling where inaccurately labeled conversions (e.g. due to computational prediction) might lead to unexpected products being produced by engineered pathways.	Attempt to classify uncertainty if possible to better inform decisions and understand the range of possible outcomes.
Uncertain completeness of source data	Underlying data are of an uncertain completeness and have missing values that causes biased results.	Whole-cell models which attempt to use all the data available, but which may be limited. Protein design often builds on sequences on those proteins so far seen, which may bias design software.	Enrich data sets with missing data or attempt to correct for known biases.
Integration of incompatible data	Data of different types and/or sources are being used together that may not be compatible with each other.	Models that need to integrate information about many different processes in a cell.	Convert data to compatible format where possible of collect complementary data that is compatible.
Capable of ecological harm	This technology has the potential to cause broad ecological harm, even if used correctly.	Gene drives used to cause extinction events and *in situ* engineering of microbiomes.	Ensure sufficient physical containment to avoid unexpected release and barriers in place if deployed.
Potential experimental hazard	Translating technology into experimental practice can require safety precautions	Toxin production, virus-like particles, work with potentially pathogenic microbes.	Assess possible safety issues and put in place necessary safety measures.

### 3.3 Case study 1: de novo protein design

**Figure 2.**
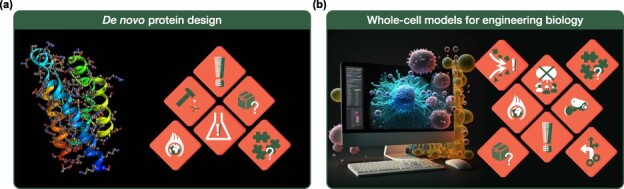
Data hazards identified for the synthetic biology case studies. (a) Data hazards associated with *de novo* protein design. Protein design is intrinsically affected by the data used to inform models and approaches, particularly from ‘incompleteness of data’: proteins that work are largely the ones selected by evolution, which are only a small fraction of what is possible. Traditional design methods rely on physical description of proteins as three-dimensional objects, but more recent data-intensive approaches operate on a much less intuitive level, i.e. often ‘harder to understand for non-experts’. These methods can also involve millions of parameters, resulting in a ‘high environmental energy cost’ for training. As proteins are some of the most versatile molecules in biology and medicine, there is a great ‘potential for misuse’ of data and design outcomes, and, for certain designs, care should be taken to ‘evaluate experimental risks’ when moving from the digital world to the laboratory. (b) Data hazards associated with whole-cell models for engineering biology. Whole-cell models rely on ‘integrating diverse and potentially incompatible data’, as well as battling with ‘gaps in the data’ that is available. The scale of these models means that they often have a ‘high environmental cost’, both in terms of their computational execution and the storage of results. This scale and complexity also makes it ‘impossible to fully understand’ how they work, making verification of their predictions and results difficult; especially as many of the modeled processes cannot be directly observed. With human whole-cell models a long-term goal of the field, their future use could open up ‘privacy concerns’ whereby models are tailored using personal information and potentially then used to ‘automate decisions’ related to treatment of disease. Furthermore, whole-cell models have been so-far been developed by a relatively ‘small community with little community input’, while their use is potentially broad with wide impact across the entire field.

To demonstrate how the data hazards framework applies to different areas of synthetic biology, we began by exploring the use of data-centric approaches for *de novo* protein design (Figure 2a). The ability to effectively design new proteins has tremendous potential for applications across numerous fields: from catalysis via novel or engineered enzymes to the sensing of molecules and synthesis of new materials. Due to the many degrees of freedom within protein chains and the complexity of the interactions involved, computational methods have been entrenched in the protein design field as early as the 1980s, first based on physicochemical principles (such as molecular dynamics) and requiring a high level of expertise to execute. The turn of the century saw the usage of optimization algorithms combining constraints and statistics-based terms. While these methods were more accessible than physics-based methods, they were often slow and didn’t capture all the relevant features of proteins (42).

Recently, as with many other areas of synthetic biology, there has been a surge in the application of machine learning approaches. The need for faster and more accurate design protocols, and the large protein datasets now available because of high-throughput sequencing and structural biology techniques has provided the impetus for data-centric approaches in nearly all aspects of protein design. Neural networks, such as AlphaFold (12) and RoseTTAFold (43), and language models, such as OmegaFold (44) and ESMFold (45), initially developed for protein structure prediction, have led to the development of new protocols with increased speed and accuracy for protein–protein and protein–ligand interaction. The availability of such tools coupled with the accessibility of cloud-based computing resources is leading to the democratization of various aspects of protein design (46). While this democratization is overwhelmingly positive for the scientific community and society at large, we should be mindful of the potential risks that this ease of use brings.

One extreme example of potential misuse is the design of protein toxins, which prompted calls for regulation already in the early 2000s (47). Protein toxins that occur naturally are highly effective at interfering with cell activity. These toxins can alter the production or breakdown of molecules involved in metabolism, degrade enzymes, or cause cell lysis by creating unregulated pores (48). The difficulty of extracting toxin samples at a scale has prevented potential misuse. However, with the aforementioned methods, one can, in principle, not only design *de novo* toxins that could readily be synthesized at a scale, but also optimize them for greater affinity and specificity. For example, having access to how various protein complexes form in humans and different proteins interact in such complexes (49) could be used to identify candidates. One can then design a toxin that interferes with the natural life cycle of those candidates. Depending on the strategy, a combination of readily available deep learning toolkits can be used [e.g. quick design of protein degraders (50)]. It is precisely due to this wide availability of easy-to-use tools that we must carefully consider the potential for misuse and how it can be mitigated.

While different data-centric protein design projects may require bespoke mitigation strategies, it is also crucial to adopt general strategies (51). One of the most important is the awareness of the problem. On an individual level, this could mean adding a brief summary of the data hazards in the data availability section of manuscripts. For a more concerted effort, we should use any conference opportunities to discuss effective long-term solutions. This would be necessary to solve any implementation details of a practical strategy such as putting any data, including model parameters, behind access schemes. Such a strategy may require new technological infrastructure to be built and maintained to maximize the accessibility of good faith individuals whilst limiting any potential misuse.

### 3.4 Case study 2: whole-cell models for engineering biology

During the first decade of the 21st century, the total amount of genome sequence data being produced doubled approximately every 7 months (52). This trend has since continued, making genomics one of the largest domains within data science, with between 2 and 40 billion gigabytes of data expected to be produced annually by 2025 (52). Unlike other big data domains, which involve mostly centralized data, genome data acquisition is highly distributed across different countries, universities, and other research laboratories. This has resulted in large quantities of often heterogeneous data, which may not always be compatible.

Whole-cell computational models (WCMs) have emerged as state-of-the-art tools for integrating vast quantities of heterogeneous sequence data, generated by high-throughput measurement techniques, into a single knowledge base for a given organism (53). This unification process involves the curation of decades’ worth of primary literature and experimental databases for determining parameter values, including protein half-lives, translation efficiencies and metabolic reaction constraints, to name just a few. The first model of this type was developed for *Mycoplasma genitalium* (18). More recently, work utilizing a more advanced WCM of *Escherichia coli*, which encompasses 19 119 parameters linked mechanistically by more than 10 000 interdependent mathematical equations (54). These parameters were extracted via a “deep curation” process that used over 400 publications spanning six decades and covering three lab strains of *E. coli*. The model comprises several sub-models, each focusing on distinct cellular processes, that are interconnected through shared resources and parameters, enabling a holistic representation of cellular dynamics under different environmental conditions. Simulating this model involves solving several types of mathematical equations simultaneously, such as ordinary differential equations, stochastic processes, and statistical models, producing over 200 000 time-series as output. The complexity of the model requires high-performance computing for execution, which may cause inequality of access, but may also beneficially act as a barrier against misuse. The intricacy of WCMs also complicates comprehension and interpretation of their output, which may discourage or limit community involvement. However, efforts to aid interpretability and visualization are being developed (55).

Within this complexity lies valuable predictive power that is being harnessed to design and conduct experiments *in silico*, accelerating scientific discovery (56–58). As WCMs become more complete and accurate, and genome engineering and synthesis become widely accessible, model-based genome design opens tremendous opportunities for the rapid engineering of biology for numerous types of application (59, 60). Utilizing WCMs in this way, however, has the potential to reinforce existing biases in the data used for their derivation, making experimental validation crucial.

The increased predictive power of WCMs compared to smaller-scale models, along with their ability to describe emergent behavior, facilitates more complex bioengineering tasks and could significantly accelerate synthetic biology design cycles. However, automated decision-making in this context may inadvertently introduce biases, potentially compromising the safety and efficacy of engineered biological systems. For example, the engineering of immune cells is currently being explored as a possible route toward novel cancer therapeutics (61). Whole-cell models of human cells (62, 63) could provide a foundation on which to build this technology by enabling efficient *in silico* assessment of different cellular reprogramming strategies. However, as highlighted in numerous medical case studies of automated decision-making using AI, the inherent biases present in medical datasets commonly used to train models or fit parameters are often not representative of the full range of demographics on which the therapeutic may be used (64). This bias could lead to the development of WCMs that aid the automated design of personalized therapies with a narrow operating window that have the potential to cause harm to subsets of the population. In addition to issues related to the application of WCMs, the high computational cost of running these large-scale simulations also has major environmental implications. For this reason, there are currently efforts to develop surrogate models which could help reduce computational burden and environmental impacts of such models (65).

Despite there only being a few WCMs created to date, the decreasing cost of genome sequencing, coupled with the exponential growth in computational power, is driving the development of WCMs for a variety of organisms. Recently developed kinetic WCMs developed for artificial cells aim to capture spatial features (e.g. they can account for cell geometry and ribosome distribution) (66). Such a variety of models may facilitate the design of pathogens or drug-resistant organisms for nefarious purposes. It is therefore essential that we adapt and extend existing synthetic biology safety frameworks to cater for the more predictive and capable design that WCMs support.

The Synthetic Biology Open Language (SBOL) serves as a prime example of a framework designed for standardizing the exchange of information related to biological designs (67). SBOL allows a user to more explicitly capture, not only structural data covering the DNA, RNA, proteins, and other chemical components within a design, but also information related to the functional interactions between these elements. The functional information is crucial for automating the development of models and in the context of WCMs, enables our knowledge of how biological processes are interwoven to be explicitly embedded. In the context of biosafety, capturing this information ensures that anyone assessing a design can more clearly see the potential for issues as less domain specific knowledge is hidden, allowing for more thorough testing of a model for potentially undesirable phenotypes. Furthermore, testing is assisted by simplified exchange of this information (68, 69). Beyond the underlying data, a sister standard called SBOL Visual (70) also offers a means to visualize biological designs in an explicit way, more clearly conveying information embedded within the SBOL data files (71). By improving the communication of intent in engineered biological designs, it is possible for potential issues to be more easily captured as less domain-specific knowledge is left with the designer. Extending both the SBOL data and visual standards to simplify the description of WCMs and the diverse processes they are required to include (e.g. making it easier to capture spatial elements and interactions between processes) would support many of these benefits.

Another example of an existing framework that could be adapted or expanded to enhance the safety of deploying WCM-designed organisms is the set of safety policies laid out by the International Genetically Engineered Machine (iGEM) Foundation (https://responsibility.igem.org/safety-policies/introduction). These focus on how teams should work during the iGEM competition (72, 73), but could provide broader guidelines that the entire synthetic biology community could choose to follow. In relation to WCMs, a new step could be introduced before the “release beyond containment” policy. This additional step would require that proper and rigorous analysis of *in-silico*-designed organisms had been performed to ensure their safety and functionality in controlled environments prior to any deployment into the physical world. Such screening is becoming common place for DNA synthesis, but has yet to be adapted more broadly for other areas of design in synthetic biology.

Constructing a whole-cell dynamical model for human cells is a central goal within systems biology (63). Developing and utilizing such models, however, raises privacy concerns, as they will likely necessitate the processing and storage of human genetic information, potentially exposing individuals to risks of unauthorized access, misuse, or discrimination. As an extreme example, such models could inadvertently publicize a given population’s genetic information, enabling someone to develop biological agents able to target specific genetic profiles. Transparency and interpretability within existing whole-cell modeling techniques, coupled with rigorous data privacy measures, will lay the foundations for safer and more reliable practices in the future by fostering a comprehensive understanding of the underlying assumptions, methodologies, and limitations of these models, as well as facilitating open and constructive scientific dialogue.

A summary of all of the hazards highlighted for using WCMs to engineer biology is shown in Figure 2b.

## 4. Discussion

Data science and AI have become increasingly popular in the field of synthetic biology as they enable new solutions to complex biological problems that would be difficult to solve otherwise. In this work, we have introduced the “Data Hazards” framework, which aims to broaden engagement in the responsible and ethical use of data science in the context of synthetic biology (Figure 1). Data Hazards is a relatively new initiative and as such is still evolving as it becomes established across different areas of science, engineering, and the humanities. Here, we identified five additional data hazards that are common in data-centric approaches to synthetic biology and used case studies covering *de novo* protein design and WCMs as a means to demonstrate how these hazards apply to emerging areas of biological engineering.

The case studies highlighted in this work (Figure 2) are only a few arbitrary examples and a much broader exercise would be needed to cover the full spectrum of potential synthetic biology research. To stimulate this process, we believe it would be valuable to consider how the use of data hazard labels could be integrated into existing scientific activities. For example, it becoming standard practice to display data hazard labels on posters at conferences or as part of graphical abstracts in papers with explanations for how these hazards have been mitigated. Such publicity would help to drive adoption and have the added benefit of establishing new ethical dialogs on research that are often lacking. Moreover, it could be beneficial to consider these hazards as research proposals are being developed to reduce the chance of misuse early on. Inclusion of a “Data Hazards Checklist” that must be completed as part of a grant application would highlight areas of concern before a project starts and ensure financial support is available to put safeguards in place or weed out research that should not be pursued due to issues that cannot be mitigated.

More broadly, the need to build a community within synthetic biology around data hazards and approaches to overcome data science risks is something that we believe could greatly benefit the field. Synthetic biology has historically been proactive about ethical considerations to ensure the benefits engineering biology offers are acceptable and understood by society and benefits and risks are discussed in a balanced way (74, 75). Considering the role of data science in these broader activities would be a valuable exercise moving forward. It is also important to note that while the new data hazard labels we have developed were done so with synthetic biology in mind, the often application agnostic use of data-centric methods means they may also be of relevance to other areas of science and engineering.

An interesting future direction for this work will be to explore how the integration of data hazards into synthetic biology design and implementation workflows can link to existing regulatory frameworks and initiatives. For example, there is growing activity in the area of sequence screening to ensure the synthesis of DNA with the potential for harm is avoided (76, 77) and the application of genomics surveillance is becoming more widely considered after the COVID-19 pandemic and rise of antimicrobial resistance (78). Biofoundries are also likely to play a key role in this area, due to their ability to generate the large data sets needed for data-centric biological design, and their central role in many projects as they scale beyond proof-of-concept studies in a research lab (79). This flexibility is essential as bioengineers battle with finding the most appropriate design methodology for the problem at hand (80).

Three of the data hazards we highlighted for synthetic biology that biofoundry capabilities could provide immediate mitigating actions include: uncertain accuracy and completeness of source data, as well as integration of incompatible data. Biofoundries require that experiments are explicitly described in a machine-readable format. This helps to support better reproducibility and improves overall accuracy of the data produced. Biofoundries are also ideally placed to implement the complex protocols often needed to provide more detailed and extensive measurements of an engineered biological system. The parallel application of highly quantitative sequencing, metabolomics and proteomics methods is necessary to gain a more complete picture of a biological system’s inner workings. However, this is rarely done due to the costs involved, difficulties in processing the biological material, and the overall complexity of the various methods applied. Biofoundries could potentially alleviate this burden and provide validated workflows where missing data are avoided and measurements are taken in absolute (35, 81) or calibrated units (34, 82, 83) that can be easily integrated. Furthermore, the ability to run experiments in high throughput also enables better estimation of both technical and biological variability, helping to quantify uncertainty as part of the measurement process.

Incentivizing the use of such facilities and rigorous metrology remains a challenge; partly due to often limited access, but also because of the perceived additional effort they impose. These hurdles could be alleviated through automation within the biofoundries themselves, funding agencies pressing for facilities to support wider synthetic biology communities outside of their host institutions, and enforcing a requirement to meet minimal data collection standards for awarded grant funding. Together, these actions would not only improve the quality of research (i.e. reproducibility due to more explicit protocols that are run by machines), but would also provide a source of high-quality data able to support advanced modeling and for secondary use by wider research communities.

In summary, data science is sure to play a crucial role as synthetic biology develops. Using hazard labels can be a useful exercise for practitioners in this field as they help to proactively identify potential risks and stimulate discussions on ways to mitigate them. By encouraging open dialogue and promoting transparency, these labels can build trust between scientists, policymakers, and the public, ultimately leading to better-informed decisions on the use of data science and AI when engineering biological systems.

## Supplementary Material

ysae010_Supp

## Data Availability

The “Data Hazards” project is a community-led initiative established in 2021 by Natalie Zelenka and Nina Di Cara. Full details about the project and all materials (e.g. Data Hazard labels, teaching materials, and a self-assessment tool) are available from https://datahazards.com under a CC-BY 4.0 license. High resolution images (PDFs) of all data hazard labels discussed in this work are also available in Supplementary Material.
