# Supplementary figures and images for: Data hazards in synthetic biology
Source: Synth Biol (Oxf). 2024 Jun 21;9(1):ysae010. doi: 10.1093/synbio/ysae010 (PMC11227101; doi:10.1093/synbio/ysae010)

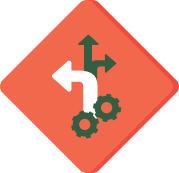

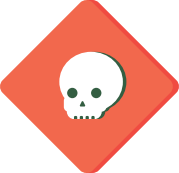

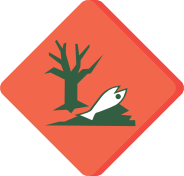

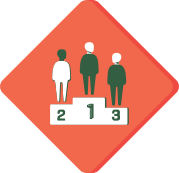

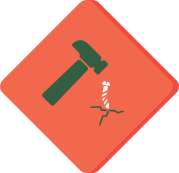

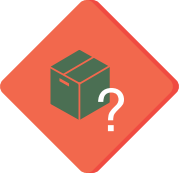

01010101  
01010101  
01010101  
01010101  
01010101  
10101010  
10101010  
10101010  
10101010  
10101010  
10101010

01010101

10101010  
10101010  
10101010

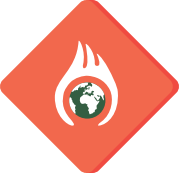

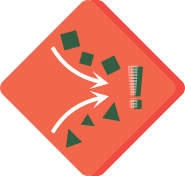

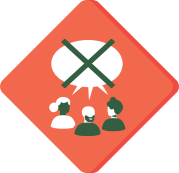

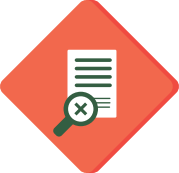

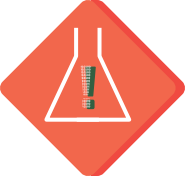

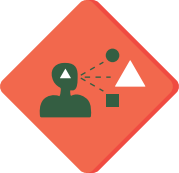

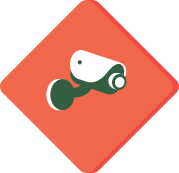

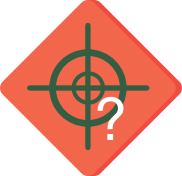

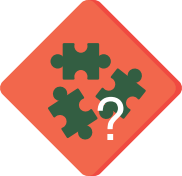

Supplement: ysae010_Supp [file ysae010_supp.zip › suppl_data/Supplementary_Material.pdf]
